# Effect of Ethanol on Branchial Adenosine Triphosphatases in *Oreochromis mossambicus* (Peters)

**DOI:** 10.4103/0971-6580.75849

**Published:** 2011

**Authors:** Smitha V. Bhanu, Babu Philip

**Affiliations:** Department of Marine Biology, Microbiology and Biochemistry, School of Marine Sciences, Cochin University of Science and Technology, Fine Arts Avenue, Cochin - 682 016, Kerala, India

**Keywords:** Analysis of variance, branchial ATPases, gill, *Oreochromis mossambicus*

## Abstract

The aim of this work was to determine the toxicity of ethanol in an aquatic system by means of bioassays with *Oreochromis mossambicus* (Peters) as a test organism. The study revealed changes in the gill ATPase activities. The results obtained indicated that ethanol brought about a decrease in the body weight, followed by significant inhibition on total ATPase, Na^+^/K^+^ ATPase, Ca^2+^ ATPase and Mg^2+^ ATPase activities. The studies also indicated that these can be employed as suitable biomarkers in ethanol related toxicity studies.

## INTRODUCTION

The use of ethanol as a fuel additive has increased the ground water contamination problems.[1] The breakdown of ethanol in surface waters through biological and chemical processes potentially results in the consumption of significant quantities of dissolved oxygen in the surface water body which will adversely affect aquatic life, resulting in fish kills.[2] Toxicants of various origins disturb the osmoregulatory potential and other physiological processes of fish.[3] The fish gill epithelium has been extensively studied as a model for ion-transporting epithelia. Gill, which is the main osmoregulatory surface tissue in aquatic animals, seems to be the primary site of uptake of waterborne pollutants; therefore, it is expected to be the first site where the sub-lethal effects of chemicals are observed.[4] ATPases are the membrane-bound enzymes concerned with immediate release of energy and are responsible for a large part of basic metabolic and physiological activities. Na^+^/K^+^ ATPase has long been studied as a target for ethanol. The activity of ATPases in fish gill represents a useful cell-membrane biomarker of pollution as it can be easily quantified.[5] Tilapia of the genus *Oreochromis* is selected as a suitable biological model for studying the mechanism of osmoregulation in teleost fish since it is capable of tolerating a wide range of salinity.[6] As there is very limited information on fish exposed to ethanol, the present study was carried out to explore the effect of ethanol on branchial ATPase activities in a fresh water fish *Oreochromis mossambicus* (Peters). An attempt has also been made to assess the possibility of using gill ATPase estimation as a suitable biomarker in ethanol related studies.

## MATERIALS AND METHODS

### Experimental design

Fresh water fish, *O. mossambicus* (Peters), commonly known as Tilapia, was selected as the animal model for the study, considering its hardy nature, ease of rearing, maintenance, availability, resistance and economic viability. They were collected from local hatcheries and acclimatized to the laboratory conditions for 15 days in large tanks filled with dechlorinated water (500 l). The tanks were previously washed with potassium permanganate to free the walls of the tanks from fungal infections. The tank had a continuous and gentle flow of tap water. The physico-chemical parameters of water were estimated daily[7] and were maintained constant throughout the experiment. The mean values for the parameters were as follows: dissolved oxygen of 8.16 ppm, total hardness 13±2 mg/l, total alkalinity 4±2 mg/l, temperature 26±2°C, pH 7.0±0.33 and salinity at 0 ppt. For conducting lethal toxicity studies, fishes were exposed to different concentrations of ethanol ranging from 1.27 to 127 g/l, in which they exhibited erratic movements, loss of equilibrium, grouping, increase in respiratory rhythm, excess secretion of mucus, followed by a gradual onset of inactivity. Each experiment was repeated three times at the selected ethanol concentration, every time noting the number of fish killed at each concentration up to 96 hours. A control without the toxicant was also maintained for both lethal and sub-lethal studies.[8] It was observed that at 13.01 g/l dose, 50% of the fishes were dead within 96 hours. The LC_50_ value for 96 hours was found to be 13.01 g/l and it was confirmed following Probit analysis.[9] From this method, it was calculated that 1.3 g/l was the sub-lethal ethanol concentration for *O. mossambicus*. To know the effect of higher concentrations as well as lower concentrations of ethanol, three sub-lethal concentrations (0.65, 1.3 and 2.6 g/l) along with control were taken as sub-lethal dose, which correspond to 1/20^th^, 1/10^th^ and 1/5^th^ of LC_50_ value, respectively.

For conducting experimental studies, *O. mossambicus* of 10±2 g were taken in three separate tubs (capacity 60 l) which contained desired concentration of ethanol (0.65, 1.3 and 2.6 g/l, respectively) along with tap water. Six replicates were kept for each experiment. A control was also maintained in the water without the addition of ethanol. Whereas in the sub-lethal toxicity study, water was changed daily and the test solutions were renewed every 24 hours to maintain the dissolved oxygen concentration at the optimum level.[10] The fishes were fed on the same commercial diet *ad libitum*. The exposure periods such as 7 and 21 days were selected as per Organization for Economic Cooperation and Development (OECD) guideline program meant for aquatic organisms.[11] During the experimental period of 21 days, the animals were fed on the same diet so as to avoid the effects of starvation on normal physiological processes and antioxidant stress. Any other factor likely to influence toxicity was nullified by maintaining a suitable control. On completion of fixed exposure period, gill tissues were dissected out. They were then washed in ice-cold 0.33 M sucrose (pH 7.5). 10% of gill homogenate was taken for the present study.

### Extraction of the enzyme

Cell fractionation of the gill homogenate was carried out according to the method of Davis (1970) with slight modifications. 10% of gill homogenate was centrifuged at 3000 *g* for 15 min in a cold refrigerated centrifuge. Supernatant was taken. It was again centrifuged at 12,000 *g* for 30 min. Clear supernatant thus obtained was taken. It was then centrifuged at 35,000 *g* for 30 min. Supernatant so obtained was discarded. The pellet obtained corresponds to the heavy microsomal fraction which was then resuspended in cold 0.33 M sucrose which served as the enzyme source. This was used for the experimental studies. They were then stored at −20°C until assayed. Immediately after thawing, gill microsomal preparations were used for the branchial ATPase activity assays. One unit of ATPase activity was expressed as micromoles of inorganic phosphate (Pi) produced by ATP decomposition per milligram protein per hour. Total ATPase activity was estimated from the amount of Pi liberated by the method of Evans.[12] Na^+^/K^+^ ATPase activity was estimated by the method of Bonting,[13] Ca^2+^ ATPase activity by the method of Hjerten and Pan[14] and Mg^2+^ ATPase activity was estimated according to the method of Ohnishi *et al*,[15] The inorganic phosphorus liberated was estimated by the method of Fiske and Subbarow.[16] The protein content of the samples was estimated by the method of Lowry *et al*.,[17] using bovine serum albumin as the standard. All the reagents used were of analytical grade.

### Statistical analysis

All processing of data was conducted with the software packages Microsoft Excel XP (for data storage) and SPSS version 15.0 (for statistical evaluation). Results are presented as mean±standard deviation (SD). Data distributions were examined to fit a normal distribution and homogeneity of variance was tested using analysis of variance (ANOVA). Data from six fish in each group were statistically analyzed adopting two-way ANOVA supplemented by multiple comparison test using Dunnett’s method. Statistical significance was accepted at *P*<0.001.

## RESULTS AND DISCUSSION

Aquatic pollutants exert a biological effect on the ATPase system by partitioning in the enzyme complex, which may cause an allosteric change that results in decreased ATPase activity.[18] Introduction of large amount of ethanol in an aquatic system results in a variety of toxicological consequences of which reduced oxygen supply is a marked respiratory effect. Biophysical studies indicate that ethanol alters membrane function by disintegrating the membrane and changing the mobility of membrane lipids and proteins. Ethanol membrane interaction specifically affects some of the membrane-bound enzymes. Fish gills being sensitive to changes are referred to as important indicators of waterborne toxicants, thereby reducing oxygen consumption and disrupting its osmoregulatory function.[19] The reduction in the activity of ATPases was correlated to the altered ionic transport and decreased ATP breakdown which had impaired the metabolic and vital physiological activities.[20]

In the present investigation, exposure of fish to 0.65, 1.3 and 2.6 g/l of ethanol for 21 days with a periodical sampling at 7 days caused marked significant (*P*<0.001) inhibition in branchial ATPase activities. Tables 1 and 2 indicate a significant decrease (*P*<0.001) in the total ATPase activity of *O. mossambicus* when exposed for 7 and 21 days, respectively, to different sub-lethal doses of ethanol. Damages in the membrane architecture may be the reason for the enzyme inhibition during the sub-lethal treatment with ethanol. Another possible reason may be the non-availability of substrates like ATP molecules, which resulted in the inhibition of ATPase. Observations made by Suhel *et al*,[21] and Jayantha *et al*,[22] support the present study.

**Table 1:** Effect of exposure to different concentrations of ethanol for 21 days on the activity of gill ATPases (micromoles of Pi liberated/hour/mg protein) in *O. mossambicus*

Parameter	Concentrations of ethanol		
	Control	0.65 g/l	1.3 g/l	2.6 g/l
Total ATPase	27.47±0.2251	23.87±0.3247	20.82±0.8414	16.20±0.1102
Na^+^/K^+^ ATPase	12.08±0.0143	9.888±0.1349	8.392±0.7664	7.096±0.1284
Ca^2^+ ATPase	7.728±0.0997	7.116±0.0333	6.248±0.1573	4.032±0.4294
Mg^2^+ ATPase	7.682±0.1070	6.827±0.1283	6.201±0.0520	5.196±0.1073

Values are Mean±SD of six observations

**Table 2:** Effect of exposure to different concentrations of ethanol for 21 days on the activity of gill ATPases (micromoles of Pi liberated/hour/mg protein) in *O. mossambicus*

Parameter	Concentrations of ethanol		
	Control	0.65 g/l	1.3 g/l	2.6 g/l
Total ATPase	26.64±0.6226	20.71±0.7307	15.51±0.1586	12.20±0.0541
Na^+^/K^+^ ATPase	11.38±0.1606	8.960±0.6726	6.670±0.4731	5.391±0.1692
Ca^2^+ ATPase	7.984±0.8211	4.760±0.3814	3.498±0.3426	2.675±0.1744
Mg^2^+ ATPase	7.110±0.0328	6.932±0.0352	5.340±0.1084	4.180±0.2537

Values are Mean±SD of six observations

The findings put forth by Simkiss[23] explain that fishes living in polluted rivers slightly impaired the main biochemical systems without causing death by resulting in the inhibition of the gill Na^+^/K^+^ ATPase activity. The present results are in agreement with previous reports stating that ethanol brings about an inhibition in Na^+^/K^+^ ATPase activity. A significant decrease (*P*<0.001) in the Na^+^/K^+^ ATPase activity [Tables 1 and 2] of *O. mossambicus* observed when exposed to 7 and 21 days mainly refers to the changes in the membrane lipid content, which in turn have been shown to influence the inhibition in Na^+^/K^+^ ATPase activity. Also, fishes entering into a state of severe hypoxia exhibited reduction in Na^+^/K^+^ ATPase activity. The findings of Kim *et al*,[24] support the present data.

Tables 1 and 2 also present a significant decrease (*P*<0.001) in the Ca^2+^ ATPase activity of *O. mossambicus* when exposed to 7 and 21 days to different sub-lethal concentrations of ethanol. Findings put forth by Ross *et al*,[25] support the present study. Mg^2+^ ATPase enzyme is always found to be associated with Na^+^/K^+^ ATPase in fish. It is also essential for the integrity of the cellular membrane and for the stabilization of branchial permeability.[26] A significant decrease (*P*<0.001) [Tables 1 and 2] observed in the Mg^2+^ ATPase activity of *O. mossambicus* when exposed for 7 and 21 days to various sub-lethal concentrations of ethanol is due to reduced ATP production which therefore results in disruption in cellular and ionic regulation. The findings of Racker *et al*,[27] support the present data. Further, ethanol suppresses the ATPase activities through activation of lipid peroxidation. The free radicals generated during the catalytic cycle of ethanol would have induced the peroxidation process in membrane lipids. The inhibition observed in the ATPase activities when *O. mossambicus* was exposed to ethanol for 21 days indicates peroxidation occurring in the damaged tissues, which brings about a change in the structure and inactivates a number of membrane-bound enzymes and protein receptors, which finally disrupts branchial membrane integrity. This statement was supported by Sato and Yonei.[28]

Two-factor ANOVA in Table 3 reveals that total ATPase, Ca^2+^ ATPase and Mg^2+^ ATPase levels varied significantly between days and between concentrations at *P*<0.001. Also, while taking into consideration the days of exposure as well as concentrations effect together (interaction), a significant difference was observed (*P*<0.001). In the case of Na^+^/K^+^ ATPase activity, significant difference (*P*<0.001) was observed between days as well as between concentrations, whereas when the effects between days of exposure as well as between concentrations were taken into account (interaction), a significant difference was observed (*P*<0.01) [Table 3]. Subsequent pair wise comparisons carried out using Dunnet’s method [Table 4] exhibited significant difference (*P*<0.001) in all the four ATPase activities when all the three sub-lethal ethanol concentrations were compared with the control.

**Table 3:** Summary of two-factor ANOVA

Parameter	Between days of exposure	Between concentrations	Days of exposure × concentration (interaction)
Total ATPase	0.000^a^	0.000^a^	0.000^a^
Na^+^/K^+^ ATPase	0.000^a^	0.000^a^	0.005^b^
Ca^2^+ ATPase	0.000^a^	0.000^a^	0.000^a^
Mg^2^+ ATPase	0.000^a^	0.000^a^	0.000^a^

The values are significant at

a *P*<0.001 and

b *P*<0.01

x= Interaction effect; 0.000 indicates that the values are significant at *P*<0.001

**Table 4:** Multiple comparison test

	Groups	Total ATPase	Na^+^/K^+^ ATPase	Ca^2+^ ATPase	Mg^2+^ ATPase
Dunnett’s	Control vs. 0.65 g/l	0.000^a^	0.000^a^	0.000^a^	0.000^a^
	Control vs. 1.3 g/l	0.000^a^	0.000^a^	0.000^a^	0.000^a^
	Control vs. 2.6 g/l	0.000^a^	0.000^a^	0.000^a^	0.000^a^

The values are significant at

a *P*<0.001

## CONCLUSION

The present findings warrant future studies to explore ATPases as possible biomarkers of ethanol related incidents in ecotoxicology. The major findings of the present experiment validate that ATPase activity can be taken as a meaningful index of cellular activity and forms a useful toxicological tool. Thus, it may be concluded by stating that fish gill can be used as a model system to study the effect of ethanol on ion transport mechanisms across cellular and epithelial membranes.
